# Heart rate variability during wakefulness reflects sleep apnea indicators but remains context-dependent

**DOI:** 10.3389/fphys.2026.1793057

**Published:** 2026-03-24

**Authors:** Paniz Balali, Ghita Omari, Amin Hossein, Elza Abdessater, Celia Batonon, Hannes Hagson, Benjamin Wacquier, Carolina Varon, Nicholas H. van den Berg, Jeroen Van Cutsem, Martine Van Puyvelde, Michael Furian, Samuel Verges, Martina Anna Maggioni, Enrico Gianluca Caiani, Olivier Debeir, Matthieu Hein, Philippe van de Borne, Nathalie Pattyn, Vitalie Faoro, Jeremy Rabineau

**Affiliations:** 1 Laboratory of Physics and Physiology (LPHYS), Département de Cardiologie, Hôpital Universitaire d’Erasme, Université libre de Bruxelles, Brussels, Belgium; 2 Laboratory of Image Synthesis and Analysis (LISA), Université Libre de Bruxelles, Brussels, Belgium; 3 Research Unit in Cardio-respiratory Physiology, Exercise & Nutrition, Faculty of Human Movement Sciences, Université libre de Bruxelles, Brussels, Belgium; 4 Concordia Research Station, French Polar Institute (IPEV), Dome C, Antarctica; 5 Service de Psychiatrie et Laboratoire du Sommeil, Hôpital Universitaire d’Erasme, Brussels, Belgium; 6 Center for Dynamical Systems, Signal Processing and Data Analytics (STADIUS), Department of Electrical Engineering (ESAT), Katholieke Universiteit Leuven (KU Leuven), Leuven, Belgium; 7 Vital Signs and Performance Research (VIPER) Research Unit, LIFE Department, Royal Military Academy, Brussels, Belgium; 8 INSERM, HP2 Laboratory, University of Grenoble Alpes, Grenoble, France; 9 Charité-Universitätsmedizin Berlin, Center for Space Medicine and Extreme Environments Berlin, Berlin, Germany; 10 Heidelberg Institute of Global Health, University Hospital, Heidelberg University, Heidelberg, Germany; 11 Department of Biomedical Sciences for Health, Università degli Studi di Milano, Milano, Italy; 12 Electronic, Information and Biomedical Engineering Department, Politecnico di Milano, Milan, Italy; 13 Department of Cardiology, Istituto di Ricovero e Cura a Carattere Scientifico (IRCCS) Istituto Auxologico Italiano, Milan, Italy; 14 Service de Psychiatrie et Laboratoire du Sommeil, Centre Hospitalier Universitaire (CHU) Brugmann, Université Libre de Bruxelles, Brussels, Belgium; 15 Brussels Laboratory of the Universe - BLU-ULB, Université libre de Bruxelles, Brussels, Belgium; 16 Department of Kinesiology and Health Sciences, University of Waterloo, Waterloo, ON, Canada

**Keywords:** autonomic regulation, extreme environments, hypobaric hypoxia, non-invasive screening, QT variability

## Abstract

Sleep-disordered breathing (SDB), particularly obstructive sleep apnea (OSA), is a major public health issue linked to cardiovascular morbidity and mortality. While polysomnography (PSG) remains the diagnostic gold standard, its complexity and cost limit widespread use. Heart rate variability (HRV) has emerged as a promising non-invasive alternative for assessing autonomic dysregulation associated with SDB, even when measured outside sleep periods. However, its reliability in extreme settings remains unclear. This study investigates whether time-domain HRV parameters measured during wakefulness reflect nocturnal SDB severity across two populations: 11 healthy men exposed to prolonged high-altitude hypoxia during a one-year stay at the Antarctic Concordia station (equivalent to ~3,800 m) and 35 clinically suspected OSA men. HRV metrics were compared against the apnea-hypopnea index (AHI) and pulse oximetry-derived respiratory indices. In the clinical group, HRV showed significant associations with OSA severity, including a negative correlation between root mean square of successive differences (RMSSD) and AHI (r = -0.524, p = 0.001) and a positive correlation between RMSSD and mean nocturnal oxygen saturation (SpO_2_) (r = 0.703, p < 0.001). In the high-altitude group, weaker but significant longitudinal associations were observed only in nights without PSG recordings, including correlations between RMSSD and SpO_2_ (r = 0.339, p = 0.016), and between deceleration capacity and SpO_2_ (r = -0.200, p = 0.009). While HRV may not serve as a definitive diagnostic marker, it could function as an early indicator of physiological stress and potential SDB, particularly in resource-limited or controlled environments. These findings underscore the need for context-specific validation of HRV-based screening tools prior to clinical implementation.

## 1 Introduction

Sleep-disordered breathing (SDB) represents a significant public health concern (Hillman et al., 2006). These disorders are broadly categorized into obstructive sleep apnea (OSA), central sleep apnea (CSA), sleep-related hypoventilation, and sleep-related hypoxemia (Tsara et al., 2009; Seet and Chung, 2017). Among these, OSA is the most prevalent, affecting almost 1 billion people globally, and with prevalence exceeding 50% in some countries (Benjafield et al., 2019). Although OSA has traditionally been reported as more prevalent in men, its prevalence in women increases substantially after menopause, approaching that of age-matched men (Bixler et al., 2001; Young et al., 2003). It is characterized by recurrent episodes of partial or complete upper airway obstruction during sleep, leading to breathing cessations lasting more than 10 seconds (Seet and Chung, 2017). CSA, on the other hand, results from the intermittent absence of central respiratory drive due to disrupted pontomedullary pacemaker activity, leading to a failure in generating neural output to activate inspiratory thoracic muscles (Selim et al., 2017). It has been shown that undiagnosed and untreated SDB is associated with increased risks of all-cause mortality, myocardial infarction, stroke, heart failure, and arrhythmias (Kendzerska et al., 2014).

Polysomnography (PSG) is the gold standard for diagnosing SDB. However, it is expensive, requires specialized infrastructure and trained personnel, and can be burdensome for patients. These factors contribute to limited accessibility and widespread underdiagnosis. To address these challenges, recent efforts have explored alternatives such as wearable and portable devices, and AI-driven diagnostic tools (Lee and Sundar, 2021). While promising, these technologies often exhibit limitations, including reduced sensitivity in mild or complex cases, poor generalizability across populations, and insufficient validation (Penzel et al., 2018).

Among these emerging approaches, the analysis of heart rate variability (HRV) derived from electrocardiogram (ECG) signals has gained attention due to its non-invasive nature and widespread availability. As cardiac autonomic activity is modulated during sleep and influenced by respiratory disturbances, HRV offers a potential window into the physiological consequences of SDB (Qin et al., 2021a; Onanga et al., 2023). Studies have demonstrated associations between HRV features and various sleep disorders, including OSA, insomnia, and excessive daytime sleepiness (Lombardi et al., 2008; Guaita et al., 2015). Reduced HRV is a well-established predictor of cardiovascular and all-cause mortality outcomes, underscoring its potential as a global biomarker of autonomic health (Jarczok et al., 2022). Nonetheless, its broader clinical use remains limited by issues such as vulnerability to confounding factors, inter-individual variability, and lack of standardized analytical protocols. In parallel, QT variability (QTV), which reflects fluctuations in ventricular repolarization (Almeida et al., 2006), has emerged as a complementary ECG-derived metric. Although less widely used than HRV, QTV may offer insights into autonomic influences on ventricular myocardial electrophysiology independent of heart rate changes, increasing with sympathetic stimulation (e.g., stress or exercise) and enabling longitudinal assessment of sympathetic activity (Magnano et al., 2002). Incorporating QTV, therefore, provides complementary insight into autonomic balance beyond what HRV alone can capture.

Because high-altitude hypoxia naturally predisposes individuals to CSA-like periodic breathing, unique environments such as the Concordia Station offer a compelling testbed to validate these autonomic markers. Located at 3,233 meters above sea level on the Antarctic Plateau (Dome C), the Concordia Station offers in fact a chronic hypobaric hypoxia that is equivalent to ~3,800 m at mid-latitudes (inspired O_2_ partial pressure ~90 mmHg vs. ~150 mmHg at sea level), which has been shown to negatively affect both sleep and cognitive performance (Van Ombergen et al., 2021). In particular, the hypobaric hypoxia at high altitude is known to promote physiological CSA, particularly in the form of periodic breathing patterns driven by respiratory control instability. Altitude-induced hyperventilation narrows the CO_2_ reserve and promotes periodic breathing depending on individual ventilatory control stability (Dempsey and Smith, 2014). Beyond altitude, Concordia also exposes crewmembers to other environmental stressors that may exacerbate SDB. The extremely low humidity, for instance, frequently leads to nasal obstruction, dry nose, and sore throat, likely due to chronic airway inflammation (Van Ombergen et al., 2021). In addition, this extreme setting disrupts circadian regulation, with crew members frequently reporting sleep disturbances related to circadian misalignment. These include reduced sleep quality (Pattyn et al., 2018), increased light sleep (Wickramasinghe and Anholm, 1999), periodic breathing (Khoo et al., 1996), and decreased performance (Titus et al., 2007), with no evidence of acclimatization even after extended stays (Porcelli et al., 2017). These conditions make Concordia a unique environment to study physiological responses to sleep disruption, especially in the absence of typical comorbidities seen in clinical populations.

In this study, we aim to evaluate whether time-domain HRV and QTV parameters described in the literature and measured during wakefulness (Qin et al., 2021a; Onanga et al., 2023) can serve as reliable indicators of SDB severity. Specifically, we ask whether these autonomic markers can consistently reflect the physiological consequences of SDB, regardless of the underlying mechanism. To address this, we compare two distinct settings: 1) a clinical dataset composed of patients suspected of having OSA and 2) a dataset collected on healthy participants experiencing mainly CSA while being involved in a one-year winter over at the Antarctic station Concordia. By examining HRV under both standard and high-altitude hypoxic conditions, we aim to assess the robustness and translational potential of these parameters across different populations and SDB patterns. We hypothesized that if HRV primarily reflects the physiological consequences of nocturnal hypoxemia rather than the underlying SDB mechanism, associations with oxygenation markers would be observable across both populations. This is particularly relevant in remote, resource-limited, or high-altitude environments, where access to traditional diagnostic tools is limited, and simplified sleep assessment methods are needed.

## 2 Materials and methods

### 2.1 Data collection (study design)

#### 2.1.1 Sea-level clinical dataset

The clinical dataset (PoSUMS study) was collected at Erasme University Hospital in Brussels, Belgium (about 20 m above sea level) as a prospective study conducted in accordance with the Declaration of Helsinki. The study received approval from the Ethics Committee of Hôpital Erasme-ULB (reference: P2023/029; B4062023000020) and is registered on ClinicalTrials.gov (identifier: NCT06029881). The dataset comprises data from participants recruited between November 2023 and December 2024. Eligible participants were male adults aged between 18 and 70 years with a body mass index (BMI) below 35 kg/m², referred by their physicians to the Sleep Laboratory at Erasme Hospital for evaluation of sleep-related complaints. Individuals with atrial fibrillation, significant valvular heart disease, or ventricular dysfunction were excluded.

**Figure 1:**
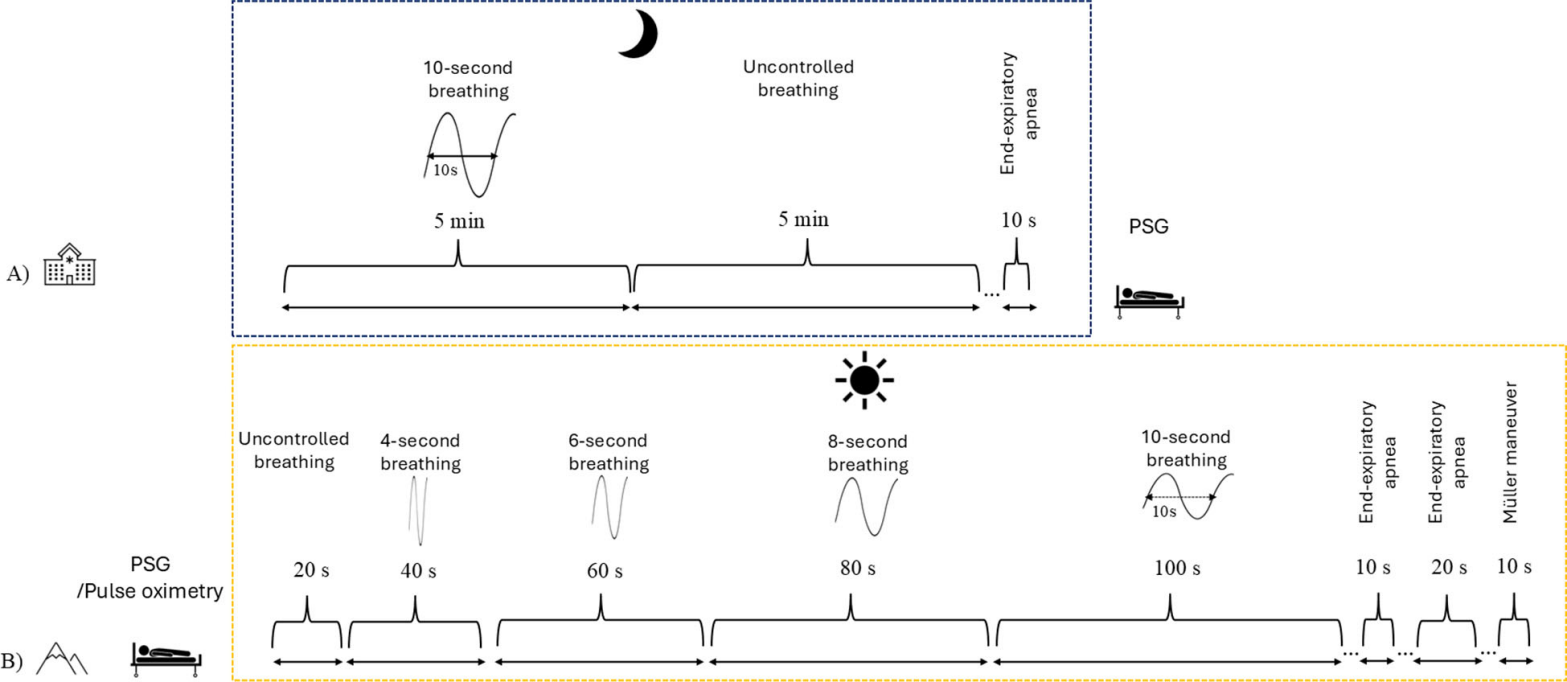
Schematic representation of study design and timing of autonomic assessments in both cohorts. **(A)** Sea-level clinical cohort: HRV measurements were performed in the evening prior to polysomnography (PSG) using a standardized respiratory protocol including 0.1 Hz paced breathing (10-second cycles), spontaneous breathing, and end-expiratory apnea. **(B)** Antarctic high-altitude cohort: Monthly overnight recordings were conducted throughout the expedition. At four predefined time points, recordings included full PSG; during the remaining months, sleep-related breathing indices were derived from pulse oximetry alone. The autonomic respiratory protocol was performed in the morning shortly after awakening and included progressively reduced breathing frequencies, end-expiratory apnea, and a Müller maneuver. For comparability, analyses were restricted to the shared 0.1 Hz paced breathing segment in both cohorts.

On the day of hospitalization, in the evening prior to PSG setup, patients were asked to perform a standardized respiratory protocol guided by an audio recording. The protocol, shown in **Figure 1**, consisted of the following phases:

-  5 minutes of paced breathing with 10-second cycles,
-  5 minutes of spontaneous (uncontrolled) breathing,
-  10-second end-expiratory apnea.

#### 2.1.2 Antarctic high-altitude dataset

The high-altitude dataset (Kinosomno study) was collected at the Concordia Station, Antarctica. On-site, one overnight ECG record was conducted each month (total: 11 planned records per participant). At 4 time points, these measurements were performed simultaneously with PSG measurements. These corresponded to the months of January, February, July, and September. When PSG was not performed concurrently with ECG, peripheral oxygen saturation (SpO_2_) was monitored using a pulse oximeter. A detailed timeline of all measurements is provided in **Table 1**.

**Table 1:** Data collection timeline at the Concordia station.

Days from arrival (mean ± SD)	N	Pulse oximetry	Polysomnography
-65 ± 17	10		
+31 ± 3	8	✔	
+57 ± 18	9		✔
+92 ± 17	8		✔
+122 ± 17	8	✔	
+157 ± 17	11	✔	
+177 ± 18	9	✔	
+206 ± 17	10	✔	
+236 ± 17	11		✔
+268 ± 18	6	✔	
+298 ± 16	11	✔	
+334 ± 17	9		✔

At baseline data collection (65 ± 17 days before arrival), only ECG data were acquired. In all cases, the heart rate variability features were evaluated in the morning, shortly after waking up. At each time point, the mean (± standard deviation) number of days before/after arrival at Concordia is indicated, while n refers to the number of subjects whose data could be collected and analyzed.

In the morning after the overnight record, the participants were asked to perform a respiratory protocol as soon as possible after waking up, normally within 15 minutes. This respiratory protocol differed from the one used in the clinical dataset. The full respiratory protocol (represented in **Figure 1**) included the following sequence:

-  20 seconds of uncontrolled breathing,
-  40 seconds of 4-second breathing cycles,
-  60 seconds of 6-second breathing cycles,
-  80 seconds of 8-second breathing cycles,
-  100 seconds of 10-second breathing cycles,
-  10 seconds of end-expiratory apnea,
-  20 seconds of end-expiratory apnea,
-  10 seconds of Müller maneuver.

Despite differences between the two protocols, both included a phase of 10-second paced breathing cycles. For comparability, we restricted our analysis to this phase in each dataset, examining a 100-second segment. This controlled deep-breathing maneuver (6 breaths per minute; 0.1 Hz) has been validated for capturing HRV patterns relevant to OSA (Onanga et al., 2023). In healthy individuals, this maneuver induces large heart rate oscillations and high vagally mediated HRV, whereas patients with OSA typically exhibit a blunted response reflecting reduced parasympathetic modulation (Onanga et al., 2023). Although originally validated in OSA, its use here provided a controlled and comparable challenge across datasets, allowing us to investigate autonomic modulation independently of the spontaneous breathing instabilities characteristic of high altitude. This maneuver maximizes respiratory sinus arrhythmia and provides robust indices of vagal activity and baroreflex sensitivity. Among standard autonomic function tests, the deep−breathing test has consistently shown the highest sensitivity for detecting autonomic dysfunction in patients with OSA, particularly in those with more severe hypoxemia (Veale et al., 1992; Izzi et al., 2018).

All procedures were conducted in accordance with the Declaration of Helsinki, and the study protocol for the Antarctic missions was approved by the European Space Agency (ESA) and by the Ethics Committee of Hôpital Erasme-ULB (P2019/301/B406201940381).

### 2.2 Participants

The clinical dataset included 35 male patients. The high-altitude data were collected from 11 male crew members who remained at the Concordia station for a duration of approximately one year. During their stay in Antarctica, participants followed their operational wake and rest schedules. The detailed demographics of each group are indicated in **Table 2**.

**Table 2:** Demographics of the subjects in both datasets.

Group	Sea-level patients	High-altitude crew	P-value	Cohen’s d
# subjects (all male)	35	11		
Age (years)	44 [37, 53]	38 [32, 54]	0.470	0.221
Body mass (kg)	89 [83, 96]	80 [73, 90]	0.037*	0.796
Body height (cm)	178 [174, 183]	177 [174, 183]	0.803	0.148
BMI	29 [26, 30]	25 [23, 29]	0.038*	0.835

Data are presented as median [first quartile; third quartile], except for the number of subjects. Values for the high-altitude dataset are based on baseline measurements taken 65 days prior to arrival at Concordia Station. * indicates p < 0.05 between groups.

All participants provided written informed consent prior to participation and were informed of their right to withdraw from the study at any time.

### 2.3 Assessments

#### 2.3.1 ECG recordings

During the respiratory protocol, ECG was recorded using the MOVESENSE Medical Sensor (Movesense, Vantaa, Finland) in the clinical dataset and using the Kino device, an in-house developed wearable (Hossein et al., 2019), in the high-altitude dataset. In both datasets, the ECG sampling rate was 500 Hz. Despite differences in the recording systems, both devices featured sensors with comparable characteristics.

#### 2.3.2 Polysomnography recordings

In both datasets, recordings were acquired using MEDATEC devices (Belgium). At Concordia Station, the Dream device was used. In the clinical dataset, recordings were obtained using either Dream device or B3IP-TC09. Each PSG session included electroencephalography (EEG), electrooculography (EOG), submental and anterior tibialis electromyography (EMG) to monitor muscle tone and limb movements, pulse oximetry, thoracoabdominal plethysmography, and airflow measurements via nasal pressure sensors or thermistors, thoracoabdominal respiratory effort belts, and a microphone, consistent with standard AASM 2012 guidelines (Iber, 2007). For the patient dataset, EEG was recorded using central (C4-M1), occipital (O2-M1), and frontal (F4-M1) derivations according to the international 10–20 system. For the high-altitude dataset, EEG was recorded using frontal derivations (Fp1-M2 and Fp2-M1).

#### 2.3.3 Pulse oximetry

Overnight peripheral oxygen saturation (SpO_2_) data were collected using either the fingertip pulse oximeter of the Dream device or a Nonin device (Nonin Medical Inc., Plymouth, Minnesota, USA) when no PSG data were acquired simultaneously.

### 2.4 Data analysis

#### 2.4.1 ECG recording

ECG signals were filtered using a bandpass filter with a range of 0.5–45 Hz. For HRV analysis, 100-second segments of 10-second breathing cycles were selected from the respiratory protocol data. In the Antarctic cohort, these 100-second segments were extracted directly from the protocol (see section 2.1.2 for detailed protocol). In the clinical dataset, the first 100 seconds during which participants adhered to the protocol were used for analysis (see the full protocol in section 2.1.1).

R peaks were detected using Kubios HRV software (version 4.1.1, Kubios Oy, Kuopio, Finland), then visually inspected from a trained operator, with manual corrections applied when necessary. From these, the RR interval time series was constructed and subsequently corrected using Kubios’s “automatic method” (Lipponen and Tarvainen, 2019). The following time-domain HRV parameters were then calculated:

- Root Mean Square of the Successive Differences (RMSSD),
- Percentage of successive normal-to-normal intervals that differ by more than 20 ms (PNN20),
- Deceleration Capacity (DC),

all of which are indicative of parasympathetic activity and tend to show reduced values in pathological conditions (Kantelhardt et al., 2007; Campana et al., 2010; Platonov and Holmqvist, 2011; Drawz et al., 2014). PNN20 was used instead of the conventional PNN50 threshold, as lower thresholds have been shown to improve sensitivity to reduced beat-to-beat variability, particularly in conditions of diminished overall HRV (Mietus et al., 2002).

ΔHR and QT variability index (QTVi) were computed using MATLAB (R2023a, MathWorks), based on the R-peaks extracted from Kubios. ΔHR was defined as the mean difference between the maximum and minimum heart rates within a respiratory cycle, as described in (Onanga et al., 2023). For this purpose, the respiration signal was derived from the ECG using the method of Varon et al (Varon et al., 2020). QTVi was computed following the approach outlined in (Baumert et al., 2016). First, the Q peak and T wave endpoints were identified beat by beat using a two-dimensional signal warping approach (Schmidt et al., 2018) to extract QT intervals. The variance normalized to the squared mean was computed for QT and RR (QTVN in and RRVN; Equations 1, 2, respectively). Finally, QTVi was computed as the logarithmic ratio of QTVN and RRVN (Equation 3).

(1) $$QTVN=\frac{QT_{var}}{QT_{{mean}^{2}}}$$

(2) $$RRVN=\frac{RR_{var}}{RR_{{mean}^{2}}}$$

(3) $$QTVi=\log\frac{QTVN}{RRVN}$$

where, QT_var_, and RR_var_ are the variances of the QT and RR intervals, and QT_mean_ and RR_mean_ their respective mean values.

#### 2.4.2 Polysomnography recording

PSG was scored in accordance with AASM guidelines (Iber, 2007). Apneic events were defined as a complete cessation of airflow lasting more than 10 seconds. Hypopneic events were characterized by a ≥30% reduction in airflow for at least 10 seconds, accompanied by a ≥4% drop in oxygen saturation (Iber, 2007). The Apnea-Hypopnea Index (AHI, events/hour) was calculated for each subject to assess the severity of SDB.

Additional PSG-derived parameters included average SpO_2_, the percentage of time spent with SpO_2_ below 90% (T90, %), and the number of oxygen desaturation events meeting a predefined amplitude criterion (e.g., ≥4% drop from baseline), typically sustained for more than 10 seconds, per hour of sleep (ODI, events/hour).

#### 2.4.3 Pulse oximetry

Pulse oximeter data was analyzed with nVISION software (version 6.5.1, Nonin Medical Inc., Plymouth, Minnesota, USA). The parameters extracted for analysis included average SpO_2_ (%), the percentage of time spent with SpO_2_ below 85% (T85, %), and the ODI (events/hour). T90 was used for the clinical dataset, whereas T85 was used for the high-altitude group to better capture desaturation severity in each context (see Limitations for details).

#### 2.4.4 Analytical considerations

Frequency-domain parameters were not computed, as the 100-second signal segments were too short to allow reliable spectral analysis. In the high-altitude dataset, reliable detection of Q-wave was not possible in several recordings due to ECG signal quality; therefore, QTVi was only computed for the clinical dataset.

### 2.5 Statistics

Statistical analyses were performed using GraphPad Prism version 9.5.1 (GraphPad Software, San Diego, CA, USA) and Python (packages: scipy.stats, pingouin, statsmodels.formula.api). In the clinical dataset, correlations between variables were assessed using Spearman analysis due to non-normal distribution of several SDB variables and potential monotonic but non-linear associations. In the high-altitude dataset, repeated measures correlation analysis (Vallat, 2018) was used to evaluate intra-individual associations across all longitudinal pulse‐oximetry sessions and, in a separate analysis, across the PSG recordings.

For the high-altitude dataset, the effect of undergoing a PSG, which can be burdensome (Lee and Sundar, 2021), on the HRV measurements was investigated. Linear mixed-effects models were fitted for each HRV feature, using PSG status as a categorical fixed effect with three levels: no PSG, first PSG night, and subsequent PSG nights. Subject was included as a random intercept to account for intra-individual variability. Model fitting was performed using the mixedlm function from the statsmodels.formula.api package. Statistical significance was defined as a p-value < 0.05. Results are presented as median [Q1; Q3], unless otherwise specified (mean ± sd).

For interpretability, effect sizes were reported as Cohen’s d. For correlation analyses, Spearman’s rho and repeated-measure correlation coefficient (r) were converted to Cohen’s d using the standard transformation (Equation 4).

(4) $$d=\frac{2r}{\sqrt{1-r^{2}}}$$

This allows interpretation of effect magnitude using conventional thresholds small (0.2≤d<0.5), moderate (0.5≤d <0.8), and large (d≥0.8).

Given the exploratory nature of the correlation analyses, no formal correction for multiple comparisons was applied. Results should therefore be interpreted with caution.

## 3 Results

### 3.1 Sea-level clinical dataset: relationship between HRV and sleep apnea indicators

**Figure 2:**
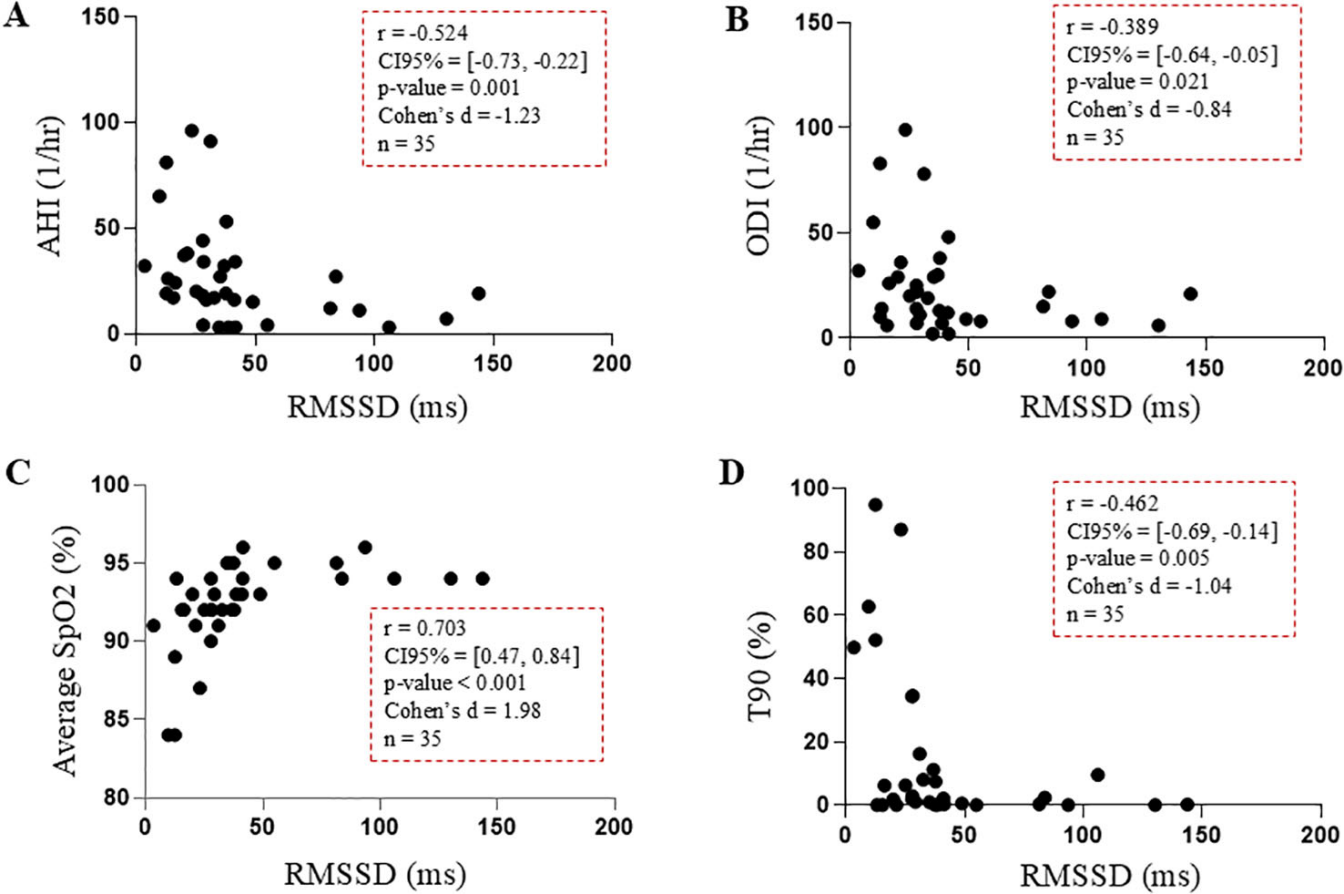
Spearman correlations between RMSSD (ms) measured during wakefulness and overnight markers of SDB in the sea-level clinical dataset: **(A)** apnea hypopnea index (AHI, 1/hr), **(B)** oxygen desaturation index (ODI, 1/hr), **(C)** average SpO_2_ (%), and **(D)** time spent with SpO_2_ < 90% (T90, %).

To evaluate the inter-individual relationship between autonomic function and SDB, we analyzed the correlations between various PSG-derived parameters collected overnight and RMSSD collected during wakefulness, as previously described (**Figure 2**). In the clinical dataset, the median RMSSD across participants was 33 [21, 41] ms. The median AHI and ODI were 19 [12, 34] and 19 [9, 30] 1/h, respectively, consisting mainly in OSA events. A breakdown of the average number of each type of apneic event (apneas only, hypopneas not shown) is provided in **Appendix 1 (Table A1)**. Median overnight SpO_2_ and T90 were 93% [92%, 94%] and 1.87% of time in bed [0.00%, 11.19%], respectively.

**Figure 2** illustrates the relationship between individual values of RMSSD during wakefulness and SDB markers. Most significant associations showed moderate-to-large effect sizes. Low values of RMSSD were correlated with high rates of apneas and desaturations (AHI: r=-0.524, CI=[-0.73, -0.22], p=0.001, and ODI: r=-0.389, CI=[-0.64, -0.05], p=0.021), as well as with low overall oxygen saturation (SpO_2_: r=0.703, CI=[0.47, 0.84], p<0.001, and T90: r=-0.462, CI=[-0.69, -0.14], p=0.005).

Similar associations were observed for HR and other parasympathetic time-domain indices measured during wakefulness (DC, PNN20, and ΔHR), see **Table 3** for complete results. QTVi was only associated with mean overnight SpO_2_ (**Table 3**).

**Table 3:** Spearman correlations coefficients between autonomic markers measured during wakefulness and SDB markers in the sea-level clinical dataset (n = 35 for all comparisons except QTVi, reported on 32 subjects due to challenges in Q wave detection in 3 participants).

SDB parameters	Autonomic markers	r	CI95%	p-value	Cohen’s d
AHI	DC	-0.52	[-0.73, -0.21]	0.001*	-1.22
	PNN20	-0.52	[-0.73, -0.22]	0.001*	-1.22
	HR	0.34	[0.00, 0.61]	0.043*	0.72
	ΔHR	-0.45	[-0.69, -0.13]	0.007*	-1.01
	QTVi	0.28	[-0.09, 0.58]	0.126	0.58
ODI	DC	-0.37	[-0.63, -0.03]	0.028*	-0.80
	PNN20	-0.39	[-0.65, -0.06]	0.020*	-0.85
	HR	0.26	[-0.09, 0.56]	0.126	0.54
	ΔHR	-0.34	[-0.61, 0.00]	0.044*	-0.72
	QTVi	0.19	[-0.18, 0.51]	0.301	0.39
Average SpO2	DC	0.66	[0.41, 0.82]	<0.001*	1.76
	PNN20	0.71	[0.48, 0.85]	<0.001*	2.02
	HR	-0.29	[-0.57, 0.06]	0.095	-0.61
	ΔHR	0.67	[0.42, 0.82]	<0.001*	1.81
	QTvi	-0.43	[-0.68, -0.09]	0.014*	-0.95
T90	DC	-0.42	[-0.67, -0.09]	0.013*	-0.93
	PNN20	-0.49	[-0.71, -0.17]	0.003*	-1.12
	HR	0.33	[-0.02, 0.61]	0.054	0.70
	ΔHR	-0.37	[-0.64, 0.03]	0.030*	-0.80
	QTVi	0.32	[-0.04, 0.61]	0.069	0.68

SDB markers: Apnea hypopnea index (AHI, 1/hr), oxygen desaturation index (ODI, 1/hr), average SpO2 (%), and time spent with SpO_2_ < 90% (T90, %). Autonomic markers: DC (ms), PNN20 (%), HR (bpm), ΔHR (bpm), and QTVi (-). *indicates p < 0.05.

### 3.2 Antarctic high-altitude dataset: relationship between HRV and sleep apnea indicators

#### 3.2.1 Nights with ECG and pulse oximetry

When analyzing longitudinal changes over time at high altitude, we found that SDB indicators remained relatively stable throughout the nights with only ECG and pulse oximetry. Overall mean (± sd) SDB-related parameters derived from pulse oximetry were as follows: ODI = 41 ± 21 1/h, SpO_2_ = 85 ± 1%, T85 = 43 ± 20%.

The monthly evolution of the HRV-related features measured during wakefulness is presented in **Appendix 2 (Table A2)**. For the nights with only ECG and pulse oximetry, these features also showed minimal night-to-night variation (mean ± sd): RMSSD = 45 ± 8 ms, DC = 60 ± 27 ms, PNN20 = 50 ± 16%, HR = 69 ± 6 bpm, and ΔHR = 15 ± 6 bpm.

**Figure 3:**
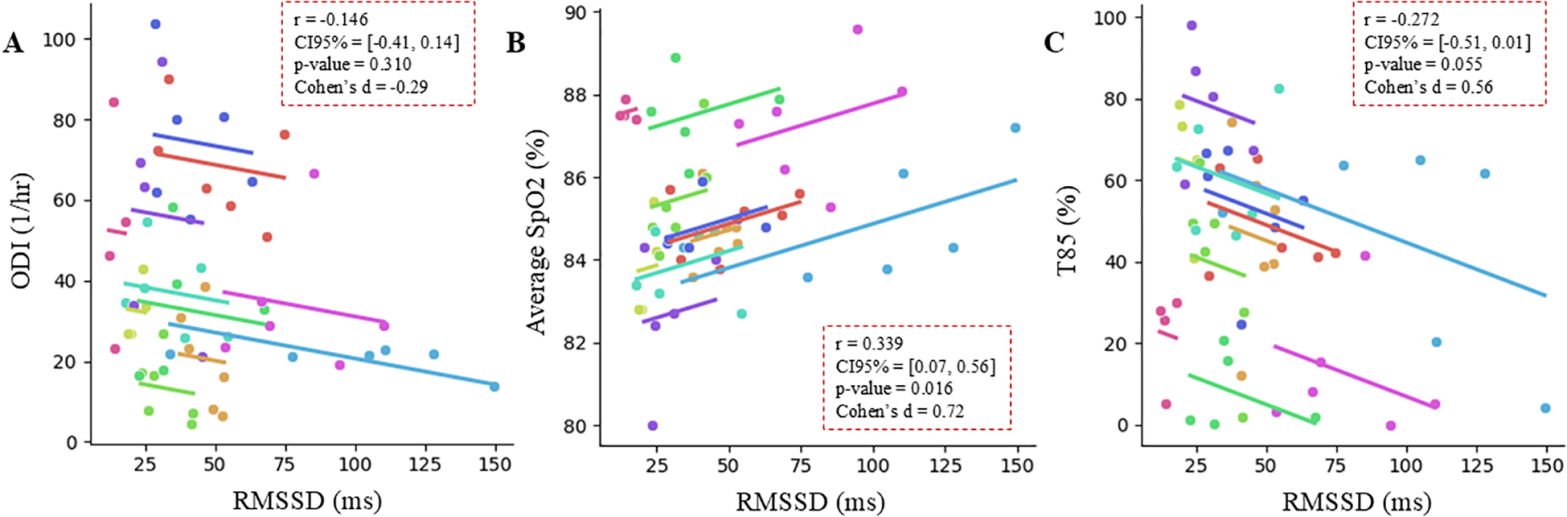
Repeated measures correlation between RMSSD (ms) measured during wakefulness and pulse oximetry-derived markers collected overnight: **(A)** oxygen desaturation index (ODI, 1/hr), **(B)** average SpO_2_ (%), and **(C)** time spent with SpO_2_ < 85% (T85, %). Data from n = 11 subjects at high altitude; mean number of timepoints per subject = 6 ± 1. Each subject is represented by a different color. The slope of the parallel lines represents the best-fit regression line slope across time points between subjects, and the length of each parallel line is constrained to the bounds of each subject’s response range.

Using pulse oximetry as a reference, the changes in RMSSD measured during wakefulness showed a tendency for associations with the associated changes in overnight mean SpO_2_ and T85 but not in ODI (p-values: 0.016, 0.055, and 0.310, respectively), as shown in **Figure 3**.

**Table 4** presents the results for repeated measures correlations between SpO_2_-derived SDB markers and the other ECG-derived autonomic markers reflecting parasympathetic activity. None of these ECG-derived features were associated with ODI, while DC and HR were significantly associated with mean overnight SpO_2_ and T85 (see **Table 4** for detailed statistics).

**Table 4:** Repeated measures correlation between overnight markers of sleep apnea, oxygen desaturation index (ODI, 1/hr), average SpO_2_ (SpO2, %), time spent with SpO_2_ < 85% (T85, %), and markers of parasympathetic activity measured during wakefulness in the high-altitude dataset: DC (ms), PNN20 (%), HR (bpm), and ΔHR (bpm).

SDB parameters	Autonomic markers	r	CI95%	p-value	Cohen’s d
ODI	DC	-0.20	[-0.45, 0.08]	0.163	-0.41
	PNN20	-0.01	[-0.29, 0.26]	0.919	-0.02
	HR	0.14	[-0.14, 0.41]	0.316	0.28
	ΔHR	-0.01	[-0.28, 0.27]	0.968	-0.02
Average SpO2	DC	0.36	[0.09, 0.58]	0.009*	0.77
	PNN20	-0.09	[-0.11, 0.43]	0.222	-0.18
	HR	-0.39	[-0.60, -0.12]	0.005*	-0.85
	ΔHR	-0.11	[-0.38, 0.17]	0.441	-0.22
T85	DC	-0.33	[-0.56, -0.06]	0.019*	-0.70
	PNN20	-0.16	[-0.42, 0.12]	0.260	-0.32
	HR	0.36	[0.10, 0.58]	0.009*	0.77
	ΔHR	0.16	[-0.13, 0.42]	0.272	0.32

*indicates p < 0.05.

#### 3.2.2 Nights with PSG

When comparing no PSG, first PSG night, and subsequent PSG nights using linear mixed-effects models, we found that HRV metrics were altered during PSG nights, particularly on the first night with PSG. Specifically, mean HR was higher (p = 0.018), while ΔHR, RMSSD, and DC were lower on the first PSG night compared to nights without PSG (p = 0.019, p = 0.021, and p = 0.033, respectively).

After excluding these familiarization nights, SDB markers remained stable across the nights assessed with PSG, with the following mean ± sd values: SpO_2_ = 84 ± 2%, AHI = 35 ± 25 1/hr, consisting mainly in CSA events. Further details on the distribution of apneic event types, limited to apneas, can be found in **Appendix 2 (Table A1)**.

HRV parameters also showed relatively constant values: RMSSD = 50 ± 38 ms, DC = 67 ± 48 ms, PNN20 = 55 ± 18%, HR = 67 ± 7 bpm, and ΔHR = 15 ± 6 bpm.

For the analysis of repeated measures correlations, the familiarization nights were excluded. Unlike SpO_2_ measured with the standalone nighttime pulse oximeter, the previously observed correlations between HRV features and average SpO2 were no longer present when examining longitudinal changes over time at high altitude. Similarly, as presented in **Table 5**, no statistically significant correlations were observed between changes in AHI and those in the time-domain HRV features reflecting parasympathetic activity.

**Table 5:** Repeated measures correlation of apnea hypopnea index (AHI, 1/hr) and average SpO_2_ (%) with markers of parasympathetic activity measured during wakefulness: RMSSD (ms), DC (ms), PNN20 (%), HR (bpm), and ΔHR (bpm).

SDB parameters	Autonomic markers	R	CI95%	p-value	Cohen’s d
AHI	RMSSD	0.12	[-0.40, 0.58]	0.667	0.24
	DC	0.10	[-0.41, 0.57]	0.707	0.20
	PNN20	0.45	[-0.06, 0.77]	0.079	1.01
	HR	-0.22	[-0.64, 0.31]	0.414	-0.45
	ΔHR	0.17	[-0.36, 0.61]	0.532	0.35
Average SpO2	RMSSD	-0.10	[-0.57, 0.42]	0.722	-0.20
	DC	-0.10	[-0.56, 0.43]	0.760	-0.20
	PNN20	-0.44	[-0.77, 0.06]	0.084	-0.98
	HR	0.32	[-0.21, 0.70]	0.229	0.68
	ΔHR	-0.10	[-0.56, 0.42]	0.736	-0.20

*indicates p < 0.05. Data from n = 11 subjects at high altitude; mean number of timepoints per subject = 3 ± 1.

## 4 Discussion

Across both datasets, sea-level patients and Antarctic high-altitude crew, reduced parasympathetic activity during wakefulness, reflected by lower time-domain HRV metrics, was associated with more severe SDB and lower nocturnal oxygen saturation. In the clinical sea-level dataset, HRV metrics such as RMSSD, DC, and PNN20 showed strong cross-sectional correlations with AHI, ODI, mean SpO_2_, and T90. In the Antarctic high-altitude dataset, HRV parameters remained relatively stable over time but demonstrated moderate associations with longitudinal changes in oxygenation indices derived from pulse oximetry, especially following nights with no PSG.

### 4.1 Association between HRV and sleep apnea indicators

The results in the clinical dataset demonstrated a consistent correlation between all HRV parameters reflecting parasympathetic activity, QTVi (a marker of sympathetic activity, less influenced by heart rate), and PSG-derived markers of SDB. These findings are in line with previous work, including a large multicentric study involving over 1247 subjects across 7 sleep centres Qin et al., 2021a), and with established evidence showing that OSA is characterized by parasympathetic withdrawal and sympathetic overactivity during wakefulness (Somers et al., 1995; Narkiewicz et al., 1998; Somers et al., 2008). Similar trends were also observed in the high-altitude dataset, where repeated-measures correlations between HRV and pulse oximetry supported these associations.

The observed reduction in HRV markers appears to be more closely linked to intermittent hypoxemia than to the number of discrete respiratory events. Traditional parameters like AHI are limited in this regard, as they cannot capture how deep or how long oxygen drops last, or the full toll they take on the body. Hypoxic burden (*i.e.*, area under each desaturation curve) was originally developed as an OSA-specific metric (Azarbarzin et al., 2019) but is increasingly recognized as clinically and prognostically meaningful in CSA as well (Oldenburg et al., 2021), providing a more comprehensive picture of the overall stress caused by disordered breathing (Azarbarzin et al., 2019). Although hypoxic burden was not computed in this analysis, the stronger association of parasympathetic HRV markers with SpO_2_-based metrics than with AHI suggests that the depth and duration of desaturations may be more relevant to autonomic disruption than event frequency alone. Future studies may benefit from including hypoxic burden to better capture these effects.

### 4.2 Inter- and intra-subject variability

Although acute exposure to high altitude is associated with marked autonomic changes, characterized by increased sympathetic activity and reduced parasympathetic modulation (Hainsworth et al., 2007; Roche et al., 2025), studies have shown that these responses only partially normalize over time. In lowlanders residing at high altitude, RMSSD and other HRV indices, after their initial reduction upon ascent, tend to recover partially but seldom return to sea-level values, even after prolonged exposure lasting several weeks or months (Dhar et al., 2014). This pattern suggests a persistent autonomic shift likely driven by the chronic hypoxic load rather than transient hypoxemia. Individual differences in habitability, chemosensitivity, ventilatory control, and sleep quality may further modulate this shift (Ainslie and Subudhi, 2014).

The absence of strong correlations between repeated HRV measures and indices such as AHI or SpO_2_ may also suggest ongoing longer-term compensatory adaptations. One such adaptation is the progressive increase in red blood cell mass, which enhances oxygen-carrying capacity and mitigates the effects of chronic hypoxemia. Classic studies show that after several months above 4,000 m, red blood cell mass and total blood volume increase substantially compared to sea level (Pugh, 1964). In addition, circadian misalignment caused by the prolonged absence of normal light-dark cycles in Antarctica (Pattyn et al., 2017) may further alter autonomic regulation, particularly by affecting parasympathetic tone (Boudreau et al., 2013). Together, these factors may blunt the acute physiological signatures typically associated with SDB, thereby weakening correlations between HRV and nocturnal desaturation metrics.

### 4.3 Potential confounding factors

In section 3.2, we observed that HRV values during the first PSG measurement, conducted two months (57 days) after arrival were significantly different from those recorded on nights without PSG, whereas no such differences were observed for the other PSG nights. This was expected, as the first-night effect (Tamaki et al., 2005), caused by the stress and increased wakefulness during an initial PSG recording, can temporarily alter autonomic markers.

Interestingly, HRV values recorded from subsequent PSG nights did not consistently reflect changes in oxygen saturation. This may be due to the fact that PSG nights inherently differ from non-instrumented nights because of the physiological and mechanical burden associated with the recording setup. We hypothesize that this may be driven by multiple stressors, including the discomfort associated with the PSG setup, combined with hypoxia-induced physiological strain and disrupted sleep architecture. These disturbances, common at high altitude, include frequent arousals, periodic breathing, and reductions in REM and deep NREM sleep (Guo et al., 2024). One possible explanation is that individuals may approach the limits of their autonomic flexibility (“HRV reserve”) under such conditions, where chronic sleep fragmentation and altered sleep stages at high altitude, compounded by PSG-related discomfort and hypoxia, leave little residual adaptive capacity. A similar phenomenon was reported in a recent crossover study at 4,000 m, where suppressing nocturnal periodic breathing with inspiratory CO_2_ did not reduce sympathetic activation or heart rate compared with unmodified breathing, indicating that hypobaric hypoxia itself imposes such a dominant autonomic load that additional SDB contributes little further (Roche et al., 2025). Therefore, the lack of significant associations during PSG nights may reflect attenuation or masking of autonomic coupling under these recording conditions rather than a true absence of physiological interaction.

Individual characteristics may further modulate autonomic tone. Some studies have reported that elderly individuals with OSA exhibit significantly lower low-frequency to high-frequency ratios compared to younger counterparts, though this difference appears to be limited to nocturnal wakefulness and is not consistently observed across sleep stages (Qin et al., 2021b). Although the average age did not differ significantly between groups in our study, individuals with longstanding SDB may exhibit blunted autonomic responses compared to those with more recent onset, independently of age.

Environmental factors unique to Concordia Station must also be considered. Although the station’s internal ambient temperature is well regulated and prolonged mild cold exposure appears to have limited effects on HRV (Harinath et al., 2005), previous reports suggest that overwintering may still be associated with reduced vagal modulation and increased sympathetic activity (Kalnish et al., 2016; Maggioni et al., 2020). Isolation and confinement may further contribute to autonomic variability through psychological stress, although these effects are difficult to isolate and likely vary depending on individual resilience and group dynamics (Pagel and Choukèr, 2016; Liu et al., 2024).

Taken together, these factors highlight that HRV features reflect cumulative physiological stress but lack specificity for distinguishing between different stressors (as illustrated by the differences observed between nights with and without PSG). In extreme environments such as the Concordia station, where numerous non-respiratory stressors act concurrently, the intra-individual relationship between HRV markers and sleep apnea severity becomes difficult to discern.

Here, we focused on time-domain HRV metrics during controlled breathing to ensure methodological consistency given the relatively short duration of the recordings. Future studies may extend this approach by incorporating frequency-domain and nonlinear indices, such as temporal asymmetry measures linked to autonomic regulation (Porta et al., 2008).

It should be noted that both cohorts included only male participants. Given known sex-related differences in SDB presentation and autonomic regulation, particularly after menopause, these findings may not be directly generalizable to women and require validation in female populations.

### 4.4 Limitations

In addition to the physiological considerations discussed above, several methodological limitations should be acknowledged when interpreting the results.

First, the relatively small sample size of the high-altitude cohort increases the risk of Type II error. Consequently, non-significant associations in this group should be interpreted cautiously and not as definitive evidence of absence of physiological interaction.

Second, HRV measurements were performed in the morning in Antarctic crew members and in the evening in the clinical cohort. Given the well-established circadian variation in vagal tone (Boudreau et al., 2013), this timing difference may have contributed to some between-group differences. Evening measurements are generally associated with higher heart rates and lower HRV, compared to morning assessments, even when controlling for chronotype (Roeser et al., 2012; Vondrasek et al., 2022). However, because analyses were conducted within each cohort separately, this factor is unlikely to have affected the internal associations observed.

Third, different oxygen desaturation thresholds were used across datasets (T90 in the clinical cohort and T85 at high altitude). This distinction was necessary because, in the clinical group, SpO_2_ levels were almost always above 85%, while the high-altitude crew spent nearly 100% of the night with SpO_2_ below 90%.

Although analyses were restricted to the shared 0.1 Hz paced breathing segment, the Antarctic protocol involved a gradual ramping of respiratory frequency prior to reaching 0.1 Hz, whereas the clinical protocol transitioned more abruptly. Subtle autonomic carry-over effects from preceding respiratory dynamics cannot be entirely excluded and may have influenced absolute HRV amplitudes. In addition, the paced breathing maneuver represents a standardized autonomic challenge and therefore reflects autonomic responsiveness under controlled conditions rather than spontaneous resting autonomic tone.

Differences in physical training status and individual ventilatory chemoreflex sensitivity between cohorts were not formally assessed and may have influenced autonomic responses. In addition, the predominant SDB phenotype differed between groups (OSA in the clinical cohort versus CSA at high altitude). Although the underlying pathophysiology differs, both conditions involve intermittent hypoxemia, allowing exploration of associations between desaturation severity and autonomic markers across these distinct settings.

Two subjects did not undergo the initial familiarization night with PSG in Antarctica, and their SDB markers may therefore have been influenced by first-night effects. However, they were retained due to the limited sample size.

Operational factors may also have contributed to variability. Not all crewmembers followed the same daily schedule, and some had night shift duties. Irregular schedules and circadian disruption associated with shift work are known to alter autonomic balance, typically reducing parasympathetic activity and increasing sympathetic tone (Fink, 2020; Liu et al., 2024). To mitigate this, HRV assessments were conducted at least three days after the last night shift whenever possible.

Finally, substantial inter-individual variability in HRV was observed across both cohorts. Such variability limits the definition of universal HRV thresholds for detecting SDB and reduces the applicability of HRV as a standalone diagnostic tool at the individual level, even when population-level associations exist.

## 5 Conclusion

In conclusion, time-domain HRV measured during wakefulness is associated with nocturnal oxygenation-related markers of SDB, but these associations are strongly context-dependent. While HRV metrics show consistent associations with sleep apnea indicators in clinical settings, their interpretation in extreme environments like high-altitude requires caution. Multiple physiological and environmental stressors can significantly modulate HRV and obscure its relationship with SDB. Although HRV cannot serve as a definitive clinical marker, it can act as a valuable early warning signal, helping to prompt timely healthcare intervention and diagnosis. These findings underscore the importance of context-specific validation before HRV can be used reliably as a surrogate marker for SDB in complex or non-standard environments. Future work should explore integrating HRV into multiparametric screening approaches combining autonomic, respiratory, and oxygenation signals to improve robustness and clinical applicability.

## Data Availability

The datasets presented in this article are not readily available due to ethical and privacy restrictions. Requests to access the datasets should be directed to the corresponding author.

## References

[B1] Ainslie P. N. Subudhi A. W. (2014). Cerebral blood flow at high altitude. High altitude Med. Biol. 15, 133–140. doi: 10.1089/ham.2013.1138, PMID: 24971767

[B2] Almeida R. Gouveia S. Rocha A. P. Pueyo E. Martínez J. P. Laguna P. (2006). QT variability and HRV interactions in ECG: quantification and reliability. IEEE Trans. Biomed. Eng. 53, 1317–1329. doi: 10.1109/TBME.2006.873682, PMID: 16830936

[B3] Azarbarzin A. Sands S. A. Taranto-Montemurro L. Redline S. Wellman A. (2019). Hypoxic burden captures sleep apnoea-specific nocturnal hypoxaemia. Eur. Heart J. 40, 2989–2990. doi: 10.1093/eurheartj/ehz274, PMID: 31071210 PMC8599917

[B4] Baumert M. Porta A. Vos M. A. Malik M. Couderc J.-P. Laguna P. . (2016). QT interval variability in body surface ECG: measurement, physiological basis, and clinical value: position statement and consensus guidance endorsed by the European Heart Rhythm Association jointly with the ESC Working Group on Cardiac Cellular Electrophysiology. Europace 18, 925–944. doi: 10.1093/europace/euv405, PMID: 26823389 PMC4905605

[B5] Benjafield A. V. Ayas N. T. Eastwood P. R. Heinzer R. Ip M. S. Morrell M. J. . (2019). Estimation of the global prevalence and burden of obstructive sleep apnoea: a literature-based analysis. Lancet Respir. Med. 7, 687–698. doi: 10.1016/S2213-2600(19)30198-5, PMID: 31300334 PMC7007763

[B6] Bixler E. O. Vgontzas A. N. Lin H.-M. Ten Have T. Rein J. Vela-Bueno A. . (2001). Prevalence of sleep-disordered breathing in women: effects of gender. Am. J. Respir. Crit. Care Med. 163, 608–613. doi: 10.1164/ajrccm.163.3.9911064, PMID: 11254512

[B7] Boudreau P. Yeh W.-H. Dumont G. A. Boivin D. B. (2013). Circadian variation of heart rate variability across sleep stages. Sleep 36, 1919–1928. doi: 10.5665/sleep.3230, PMID: 24293767 PMC3825442

[B8] Campana L. M. Owens R. L. Clifford G. D. Pittman S. D. Malhotra A. (2010). Phase-rectified signal averaging as a sensitive index of autonomic changes with aging. J. Appl. Physiol. 108, 1668–1673. doi: 10.1152/japplphysiol.00013.2010, PMID: 20339014 PMC2886688

[B9] Dempsey J. A. Smith C. A. (2014). Pathophysiology of human ventilatory control. Eur. Respir. J. 44, 495–512. doi: 10.1183/09031936.00048514, PMID: 24925922 PMC4578297

[B10] Dhar P. Sharma V. K. Hota K. B. Das S. K. Hota S. K. Srivastava R. B. . (2014). Autonomic cardiovascular responses in acclimatized lowlanders on prolonged stay at high altitude: a longitudinal follow up study. PLoS One 9, e84274. doi: 10.1371/journal.pone.0084274, PMID: 24404157 PMC3880292

[B11] Drawz P. E. Babineau D. C. Brecklin C. He J. Kallem R. R. Soliman E. Z. . (2014). Heart rate variability is a predictor of mortality in chronic kidney disease: a report from the CRIC Study. Am. J. Nephrol. 38, 517–528. doi: 10.1159/000357200, PMID: 24356377 PMC3920657

[B12] Fink A. M. (2020). Measuring the effects of night-shift work on cardiac autonomic modulation: an appraisal of heart rate variability metrics. Int. J. Occup. Med. Environ. Health 33, 409–425. doi: 10.13075/ijomeh.1896.01560, PMID: 32427129

[B13] Guaita M. Melia U. Vallverdu M. Caminal P. Vilaseca I. Montserrat J. M. . (2015). Regularity of cardiac rhythm as a marker of sleepiness in sleep disordered breathing. PLoS One 10, e0122645. doi: 10.1371/journal.pone.0122645, PMID: 25860587 PMC4393025

[B14] Guo C. Lan L. Yan Y. Kang M. (2024). Effects of acute exposure to hypoxia on sleep structure in healthy adults: a systematic review. Sleep Med. Rev. 75, 101928. doi: 10.1016/j.smrv.2024.101928, PMID: 38614049

[B15] Hainsworth R. Drinkhill M. J. Rivera-Chira M. (2007). The autonomic nervous system at high altitude. Clin. Autonomic Res. 17, 13–19. doi: 10.1007/s10286-006-0395-7, PMID: 17264976 PMC1797062

[B16] Harinath K. Malhotra A. S. Pal K. Prasad R. Kumar R. Sawhney R. C. (2005). Autonomic nervous system and adrenal response to cold in man at Antarctica. Wilderness Environ. Med. 16, 81–91. doi: 10.1580/PR30-04.1, PMID: 15974257

[B17] Hillman D. R. Murphy A. S. Antic R. Pezzullo L. (2006). The economic cost of sleep disorders. Sleep 29, 299–305. doi: 10.1093/sleep/29.3.299, PMID: 16553015

[B18] Hossein A. Mirica D. C. Rabineau J. Rio J. I. D. Morra S. Gorlier D. . (2019). Accurate detection of dobutamine-induced haemodynamic changes by kino-cardiography: a randomised double-blind placebo-controlled validation study. Sci. Rep. 9, 10479. doi: 10.1038/s41598-019-46823-3, PMID: 31324831 PMC6642180

[B19] Iber C. (2007). The AASM manual for the scoring of sleep and associated events: rules, terminology, and technical specification. (Westchester, Illinois, United States: American Academy of Sleep Medicine).

[B20] Izzi F. Placidi F. Liguori C. Lauretti B. Marfia G. A. Pisani A. . (2018). Does continuous positive airway pressure treatment affect autonomic nervous system in patients with severe obstructive sleep apnea? Sleep Med. 42, 68–72. doi: 10.1016/j.sleep.2017.09.029, PMID: 29458748

[B21] Jarczok M. N. Weimer K. Braun C. Williams D. P. Thayer J. F. Guendel H. O. . (2022). Heart rate variability in the prediction of mortality: A systematic review and meta-analysis of healthy and patient populations. Neurosci. Biobehav. Rev. 143, 104907. doi: 10.1016/j.neubiorev.2022.104907, PMID: 36243195

[B22] Kalnish V. Pyshnov G. Y. Vysotska L. (2016). Heart rate regulation during adaptation to conditions in Antarctica. Fiziolohichnyi Zhurnal (Kiev Ukraine: 1994) 62, 20–29. doi: 10.15407/fz62.03.020, PMID: 29569882

[B23] Kantelhardt J. W. Bauer A. Schumann A. Y. Barthel P. Schneider R. Malik M. . (2007). Phase-rectified signal averaging for the detection of quasi-periodicities and the prediction of cardiovascular risk. Chaos: Interdiscip. J. Nonlinear Sci. 17, 015112. doi: 10.1063/1.2430636, PMID: 17411269

[B24] Kendzerska T. Mollayeva T. Gershon A. S. Leung R. S. Hawker G. Tomlinson G. (2014). Untreated obstructive sleep apnea and the risk for serious long-term adverse outcomes: a systematic review. Sleep Med. Rev. 18, 49–59. doi: 10.1016/j.smrv.2013.01.003, PMID: 23642349

[B25] Khoo M. C. Anholm J. D. Ko S.-W. Downey R. III Powles A. P. Sutton J. R. . (1996). Dynamics of periodic breathing and arousal during sleep at extreme altitude. Respiration Physiol. 103, 33–43. doi: 10.1016/0034-5687(95)00057-7, PMID: 8822221

[B26] Lee J. J. Sundar K. M. (2021). Evaluation and management of adults with obstructive sleep apnea syndrome. Lung 199, 87–101. doi: 10.1007/s00408-021-00426-w, PMID: 33713177

[B27] Lipponen J. A. Tarvainen M. P. (2019). A robust algorithm for heart rate variability time series artefact correction using novel beat classification. J. Med. Eng. Technol. 43, 173–181. doi: 10.1080/03091902.2019.1640306, PMID: 31314618

[B28] Liu S. Wang J. Chen S. Chai J. Wen J. Tian X. . (2024). Vagal predominance correlates with mood state changes of winter-over expeditioners during prolonged Antarctic residence. PloS One 19, e0298751. doi: 10.1371/journal.pone.0298751, PMID: 38968274 PMC11226091

[B29] Lombardi C. Parati G. Cortelli P. Provini F. Vetrugno R. Plazzi G. . (2008). Daytime sleepiness and neural cardiac modulation in sleep-related breathing disorders. J. sleep Res. 17, 263–270. doi: 10.1111/j.1365-2869.2008.00659.x, PMID: 18503513

[B30] Maggioni M. A. Merati G. Castiglioni P. Mendt S. Gunga H.-C. Stahn A. C. (2020). Reduced vagal modulations of heart rate during overwintering in Antarctica. Sci. Rep. 10, 21810. doi: 10.1038/s41598-020-78722-3, PMID: 33311648 PMC7733485

[B31] Magnano A. R. Holleran S. Ramakrishnan R. Reiffel J. A. Bloomfield D. M. (2002). Autonomic nervous system influences on QT interval in normal subjects. J. Am. Coll. Cardiol. 39, 1820–1826. doi: 10.1016/S0735-1097(02)01852-1, PMID: 12039498

[B32] Mietus J. Peng C. Henry I. Goldsmith R. Goldberger A. (2002). The pNNx files: re-examining a widely used heart rate variability measure. Heart 88, 378–380. doi: 10.1136/heart.88.4.378, PMID: 12231596 PMC1767394

[B33] Narkiewicz K. Montano N. Cogliati C. Van De Borne P. J. Dyken M. E. Somers V. K. (1998). Altered cardiovascular variability in obstructive sleep apnea. Circulation 98, 1071–1077. doi: 10.1161/01.CIR.98.11.1071, PMID: 9736593

[B34] Oldenburg O. Costanzo M. R. Germany R. McKane S. Meyer T. E. Fox H. (2021). Improving nocturnal hypoxemic burden with transvenous phrenic nerve stimulation for the treatment of central sleep apnea. J. Cardiovasc. Trans. Res. 14, 377–385. doi: 10.1007/s12265-020-10061-0, PMID: 32789619 PMC8043931

[B35] Onanga M. Joanny S. Rivals I. Perger E. Arnulf I. Redolfi S. . (2023). Screening of obstructive sleep apnea syndrome by the deep breathing technique. J. Clin. Sleep Med. 19, 293–302. doi: 10.5664/jcsm.10314, PMID: 36148620 PMC9892745

[B36] Pagel J. I. Choukèr A. (2016). Effects of isolation and confinement on humans-implications for manned space explorations. J. Appl. Physiol. 120, 1449–1457. doi: 10.1152/japplphysiol.00928.2015, PMID: 26846554

[B37] Pattyn N. Mairesse O. Cortoos A. Marcoen N. Neyt X. Meeusen R. (2017). Sleep during an Antarctic summer expedition: new light on “polar insomnia”. J. Appl. Physiol. 122, 788–794. doi: 10.1152/japplphysiol.00606.2016, PMID: 28082331

[B38] Pattyn N. Van Puyvelde M. Fernandez-Tellez H. Roelands B. Mairesse O. (2018). From the midnight sun to the longest night: sleep in Antarctica. Sleep Med. Rev. 37, 159–172. doi: 10.1016/j.smrv.2017.03.001, PMID: 28460798

[B39] Penzel T. Schöbel C. Fietze I. (2018). New technology to assess sleep apnea: wearables, smartphones, and accessories. F1000Research 7, 413. doi: 10.12688/f1000research.13010.1, PMID: 29707207 PMC5883394

[B40] Platonov P. G. Holmqvist F. (2011). Atrial fibrillatory rate and irregularity of ventricular response as predictors of clinical outcome in patients with atrial fibrillation. J. electrocardiology 44, 673–677. doi: 10.1016/j.jelectrocard.2011.07.024, PMID: 21907998

[B41] Porcelli S. Marzorati M. Healey B. Terraneo L. Vezzoli A. Bella S. D. . (2017). Lack of acclimatization to chronic hypoxia in humans in the Antarctica. Sci. Rep. 7, 18090. doi: 10.1038/s41598-017-18212-1, PMID: 29273712 PMC5741743

[B42] Porta A. Casali K. R. Casali A. G. Gnecchi-Ruscone T. Tobaldini E. Montano N. . (2008). Temporal asymmetries of short-term heart period variability are linked to autonomic regulation. Am. J. Physiology-Regulatory Integr. Comp. Physiol. 295, R550–R557. doi: 10.1152/ajpregu.00129.2008, PMID: 18495836

[B43] Pugh L. (1964). Blood volume and haemoglobin concentration at altitudes above 18, 000 ft.(5500 m). J. Physiol. 170, 344. doi: 10.1113/jphysiol.1964.sp007335, PMID: 14165170 PMC1368819

[B44] Qin H. Keenan B. T. Mazzotti D. R. Vaquerizo-Villar F. Kraemer J. F. Wessel N. . (2021a). Heart rate variability during wakefulness as a marker of obstructive sleep apnea severity. Sleep 44, zsab018. doi: 10.1093/sleep/zsab018, PMID: 33506267 PMC8120337

[B45] Qin H. Steenbergen N. Glos M. Wessel N. Kraemer J. F. Vaquerizo-Villar F. . (2021b). The different facets of heart rate variability in obstructive sleep apnea. Front. Psychiatry 12, 642333. doi: 10.3389/fpsyt.2021.642333, PMID: 34366907 PMC8339263

[B46] Roche J. Fisher J. P. Rasmussen P. Ibrahim A. Ainslie P. N. Turner R. . (2025). Effects of nocturnal periodic breathing on sympathetic nerve activity and ventilatory control at high altitude: a randomised, crossover study. J. Physiol. 66, 1–15. doi: 10.1183/13993003.congress-2025.OA3304, PMID: 40684423

[B47] Roeser K. Obergfell F. Meule A. Vögele C. Schlarb A. A. Kübler A. (2012). Of larks and hearts—morningness/eveningness, heart rate variability and cardiovascular stress response at different times of day. Physiol. Behav. 106, 151–157. doi: 10.1016/j.physbeh.2012.01.023, PMID: 22330324

[B48] Schmidt M. Baumert M. Malberg H. Zaunseder S. (2018). Iterative two-dimensional signal warping—Towards a generalized approach for adaption of one-dimensional signals. Biomed. Signal Process. Control 43, 311–319. doi: 10.1016/j.bspc.2018.03.016, PMID: 41853590

[B49] Seet E. Chung F. (2017). “ Preoperative, perioperative, and postoperative evaluation and management of sleep-disordered breathing,” in Sleep disorders medicine: basic science, technical considerations and clinical aspects (New York, NY, United States: Springer) 647–659.

[B50] Selim B. J. Somers V. Caples S. M. (2017). “ Central sleep apnea, hypoventilation syndrome, and sleep in high altitude,” in Sleep disorders medicine: basic science, technical considerations and clinical aspects (New York, NY, United States: Springer), 597–618.

[B51] Somers V. K. Dyken M. E. Clary M. P. Abboud F. M. (1995). Sympathetic neural mechanisms in obstructive sleep apnea. J. Clin. Invest. 96, 1897–1904. doi: 10.1172/JCI118235, PMID: 7560081 PMC185826

[B52] Somers V. K. White D. P. Amin R. Abraham W. T. Costa F. Culebras A. . (2008). Sleep apnea and cardiovascular disease: An American heart association/American college of cardiology foundation scientific statement from the American heart association council for high blood pressure research professional education committee, council on clinical cardiology, stroke council, and council on cardiovascular nursing in collaboration with the national heart, lung, and blood institute national center on sleep disorders research (national institutes of health). Circulation 118, 1080–1111. doi: 10.1161/CIRCULATIONAHA.107.189420, PMID: 18725495

[B53] Tamaki M. Nittono H. Hayashi M. Hori T. (2005). Examination of the first-night effect during the sleep-onset period. Sleep 28, 195–202. doi: 10.1093/sleep/28.2.195, PMID: 16171243

[B54] Titus A. Rao B. S. Harsha H. Ramkumar K. Srikumar B. Singh S. . (2007). Hypobaric hypoxia-induced dendritic atrophy of hippocampal neurons is associated with cognitive impairment in adult rats. Neuroscience 145, 265–278. doi: 10.1016/j.neuroscience.2006.11.037, PMID: 17222983

[B55] Tsara V. Amfilochiou A. Papagrigorakis M. Georgopoulos D. Liolios E. (2009). Definition and classification of sleep related breathing disorders in adults: Different types and indications for sleep studies (Part 1). Hippokratia 13, 187. 19918312 PMC2765300

[B56] Vallat R. (2018). Pingouin: statistics in python. J. Open Source Software 3, 1026. doi: 10.21105/joss.01026

[B57] Van Ombergen A. Rossiter A. Ngo-Anh T. J. (2021). ’White Mars’–nearly two decades of biomedical research at the Antarctic Concordia station. Exp. Physiol. 106, 6–17. doi: 10.1113/EP088352, PMID: 32662901

[B58] Varon C. Morales J. Lázaro J. Orini M. Deviaene M. Kontaxis S. . (2020). A comparative study of ECG-derived respiration in ambulatory monitoring using the single-lead ECG. Sci. Rep. 10, 5704. doi: 10.1038/s41598-020-62624-5, PMID: 32235865 PMC7109157

[B59] Veale D. Pepin J. Levy P. (1992). Autonomic stress tests in obstructive sleep apnea syndrome and snoring. Sleep 15, 505–513. doi: 10.1093/sleep/15.6.505, PMID: 1475565

[B60] Vondrasek J. D. Alkahtani S. A. Al-Hudaib A. A. Habib S. S. Al-Masri A. A. Grosicki G. J. . (2022). “ Heart rate variability and chronotype in young adult men,” in Healthcare (Basel, Switzerland: MDPI). 10.3390/healthcare10122465 PMC9777576 36553989

[B61] Wickramasinghe H. Anholm J. D. (1999). Sleep and breathing at high altitude. Sleep Breathing 3, 89–101. doi: 10.1007/s11325-999-0089-1, PMID: 11898114

[B62] Young T. Finn L. Austin D. Peterson A. (2003). Menopausal status and sleep-disordered breathing in the Wisconsin Sleep Cohort Study. Am. J. Respir. Crit. Care Med. 167, 1181–1185. doi: 10.1164/rccm.200209-1055OC, PMID: 12615621

