# Supplementary material for: Heart rate variability during wakefulness reflects sleep apnea indicators but remains context-dependent
Source: Front Physiol. 2026 Mar 24;17:1793057. doi: 10.3389/fphys.2026.1793057 (PMC13053222; doi:10.3389/fphys.2026.1793057)
Supplement: Supplementary file 1 [file DataSheet1.pdf]

Appendix 1

Table A1. Average number of central, obstructive, and mixed apneas per night across the two datasets. Values represent median [Q1, Q3].

	Patients	High-Altitude
Obstructive Apnea (events)	33 [7, 72]	0 [0, 3]
Central Apnea (events)	3 [0, 8]	100 [40, 252]
Mixed Apnea (events)	1 [0, 4]	5 [2, 17]

Appendix 2

*Table A2. HR and HRV results in both the patients dataset and the high-altitude dataset through time (days before/after arrival). Variables shown include: Mean HR (mean heart rate in beats per minute, bpm), RMSSD (root mean square of successive differences, ms), DC (deceleration capacity, ms), PNN20 (percentage of successive RR intervals differing by more than 20 ms, %), ΔHR (mean difference between maximum and minimum heart rates within a respiratory cycle, bpm), and QTVi (QT variability index, unitless). * indicates timepoints at which PSG was performed; ** indicates the first night with PSG.*

Dataset	Timepoint	Mean HR	RMSSD	DC	PNN20	ΔHR	QTVi
Patients	/	71 [64, 75]	32 [21, 41]	49 [29, 79]	50 [29, 70]	13 [10, 18]	-0.698 [-1.358, -0.399]
High-altitude	Baseline (-65)	67 [61, 73]	49 [28, 60]	76 [55, 84]	63 [49, 67]	14 [13, 21]	/
	December (+31)	70 [67, 72]	34 [29, 50]	47 [37, 61]	55 [47, 59]	18 [12, 20]	/
	January (+57)**	70 [69, 76]	24 [14, 32]	31 [22, 47]	32 [19, 51]	12 [8, 17]	/
	February (+93)*	65 [60, 72]	40 [22, 65]	63 [35, 73]	61 [37, 70]	15 [10, 17]	/
	March (+122)	65 [64, 72]	39 [26, 97]	53 [39, 106]	52 [45, 75]	14 [13, 26]	/
	April (+157)	69 [67, 74]	30 [23, 37]	41 [30, 60]	49 [31, 58]	12 [11, 18]	/
	May (+178)	68 [60, 79]	29 [21, 64]	43 [29, 94]	49 [34, 67]	12 [10, 20]	/
	June (+206)	64 [55, 70]	40 [23, 65]	66 [38, 88]	59 [34, 69]	11 [10, 17]	/
	July (+236)*	69 [60,74]	42 [25, 48]	52 [34, 76]	54 [49, 68]	13 [12, 17]	/

	August (+269)	64 [62, 73]	48 [34, 68]	72 [60, 88]	57 [51, 63]	15 [12, 20]	/
	September (+298)	66 [63, 69]	46, [24, 67]	50 [38,87]	56 [51, 63]	12 [10, 20]	/
	October (+334)*	65 [61, 70]	50 [32, 57]	58 [49, 92]	60 [47, 69]	15 [12, 20]	/
